# Resuscitative endovascular balloon occlusion of the aorta vs epinephrine in the treatment of non-traumatic cardiac arrest in swine

**DOI:** 10.1186/s13613-021-00871-z

**Published:** 2021-05-17

**Authors:** Alice Hutin, Yaël Levy, Fanny Lidouren, Matthias Kohlhauer, Pierre Carli, Bijan Ghaleh, Lionel Lamhaut, Renaud Tissier

**Affiliations:** 1 Univ Paris Est Créteil, INSERM, IMRB, 94010 Créteil, France; 2 Ecole Nationale Vétérinaire D’Alfort, IMRB, AfterROSC Network, 7 avenue du Général de Gaulle, 94700 Maisons-Alfort, France; 3 SAMU de Paris-ICU, Necker University Hospital, Assistance Publique-Hôpitaux de Paris, Université de Paris, 75015 Paris, France; 4 INSERM U970, PARCC, CEMS, Paris, France

**Keywords:** Resuscitative endovascular balloon occlusion of the aorta, Epinephrine, Cardiac arrest

## Abstract

**Background:**

The administration of epinephrine in the management of non-traumatic cardiac arrest remains recommended despite controversial effects on neurologic outcome. The use of resuscitative endovascular balloon occlusion of the aorta (REBOA) could be an interesting alternative. The aim of this study was to compare the effects of these 2 strategies on return of spontaneous circulation (ROSC) and cerebral hemodynamics during cardiopulmonary resuscitation (CPR) in a swine model of non-traumatic cardiac arrest.

**Results:**

Anesthetized pigs were instrumented and submitted to ventricular fibrillation. After 4 min of no-flow and 18 min of basic life support (BLS) using a mechanical CPR device, animals were randomly submitted to either REBOA or epinephrine administration before defibrillation attempts. Six animals were included in each experimental group (Epinephrine or REBOA). Hemodynamic parameters were similar in both groups during BLS, i.e., before randomization. After epinephrine administration or REBOA, mean arterial pressure, coronary and cerebral perfusion pressures similarly increased in both groups. However, carotid blood flow (CBF) and cerebral regional oxygenation saturation were significantly higher with REBOA as compared to epinephrine administration (+ 125% and + 40%, respectively). ROSC was obtained in 5 animals in both groups. After resuscitation, CBF remained lower in the epinephrine group as compared to REBOA, but it did not achieve statistical significance.

**Conclusions:**

During CPR, REBOA is as efficient as epinephrine to facilitate ROSC. Unlike epinephrine, REBOA transitorily increases cerebral blood flow and could avoid its cerebral detrimental effects during CPR. These experimental findings suggest that the use of REBOA could be beneficial in the treatment of non-traumatic cardiac arrest.

## Background

Although the use of epinephrine is recommended by international guidelines in the treatment of cardiac arrest (CA), the beneficial effects of epinephrine are questioned during advanced life support. As shown by observational data and more recently in a randomized study comparing the administration of epinephrine to placebo, epinephrine increases chances of return of spontaneous circulation (ROSC) and survival rate at 30 days but does not improve neurological outcome at hospital discharge, especially with repeated doses [1–3]. Experimental data provide some answers to these ambivalent effects of epinephrine (i.e., favorable cardiovascular vs unfavorable neurologic effects). It has indeed been shown that epinephrine administration during cardiopulmonary resuscitation (CPR) leads to an increase in mean arterial pressure (MAP) and coronary perfusion pressure (CoPP) thus facilitating ROSC [4]. However, epinephrine administration is also associated to a decrease in cerebral perfusion illustrated by decreased carotid blood flow (CBF) [5], cerebral microvascular blood flow and impaired cerebral oxygenation [6, 7].

With this in mind, other strategies are considered to avoid the administration of epinephrine during CPR. Resuscitative endovascular balloon occlusion of the aorta (REBOA) is currently used in the treatment of hemorrhagic shock or traumatic cardiac arrest [8]. More recently, the use of REBOA has also been suggested for the treatment of non-traumatic CA [9], especially after failure of conventional advanced life support [10]. Experimental data have shown that aortic occlusion during CPR increases MAP and CoPP [11] thus increasing chances of ROSC. Moreover, the inflation of the REBOA increases cerebral perfusion pressure (CePP) [12] as well as carotid blood flow [11] when combined to epinephrine. However, although clinical data are emerging, showing the feasibility of REBOA implementation during CPR [13], even in the prehospital setting [14], the effect of REBOA has not been considered as a sole replacement therapy during non-traumatic CA.

Accordingly, the goal of this study was to determine whether the effect of REBOA during CPR on cardiac afterload could be used as a substitute for epinephrine administration in non-traumatic CA, to obtain ROSC while avoiding deleterious effects of epinephrine on cerebral microcirculation. We compared the effect of REBOA to epinephrine administration on both coronary and cerebral hemodynamics in a porcine model of non-traumatic CA. In order to mimic the clinical time frame [3], the swine were submitted to a prolonged period of basic life support (BLS) (i.e., 18 min) before being allocated to advanced life support (ALS) with either epinephrine administration or REBOA.

## Methods

The protocol was approved by the French national ethical committee (ComEth Anses/EnvA/UPEC n°16, project #27365-2020092810214926).

### Animal preparation

Female pigs crossed between Large White and Landrace were anesthetized with a mixture of zolazepam/tiletamine (10 mg/kg, i.m.), and propofol (10 mg/kg, i.v.); methadone was administered for analgesia (0.3 mg/kg, i.m.). Animals were intubated and submitted to conventional mechanical ventilation (FiO_2_ = 30%; tidal volume = 8 ml/kg; respiratory rate = 18 breaths/min; Monnal T60®, Air Liquide Medical Systems, Antony, France). Ventilation parameters were adjusted to maintain normocapnia. They were then instrumented with fluid-filled catheters placed into the descending aorta and right atrium through two sheaths (9Fr) inserted into the left femoral artery and vein, respectively, in order to invasively monitor mean arterial pressure (MAP) and right atrial pressure. Coronary perfusion pressure (CoPP) was then calculated as the difference between MAP and mean right atrial pressure. During CPR, measures were made at end-decompression. A blood flow probe (PS-Series Probes, Transonic, NY, USA) was surgically placed around the carotid artery to monitor carotid blood flow (CBF). A pressure sensing catheter (Millar®, SPR-524, Houston, TX, USA) was inserted after craniotomy to monitor intracranial pressure (ICP). Cerebral perfusion pressure (CePP) was then calculated as the difference between MAP and ICP, as well as cerebral vascular resistance (CVR = CePP/CBF). Electrocardiogram (ECG) and end-tidal CO2 were continuously monitored. In order to monitor cerebral regional oxygen saturation, a Near-infrared spectroscopy (NIRS) electrode was attached to the pig’s scalp over the right hemisphere (INVOS™ 5100C Cerebral/Somatic Oximeter, Medtronic®).

### Experimental protocol

**Fig. 1:**
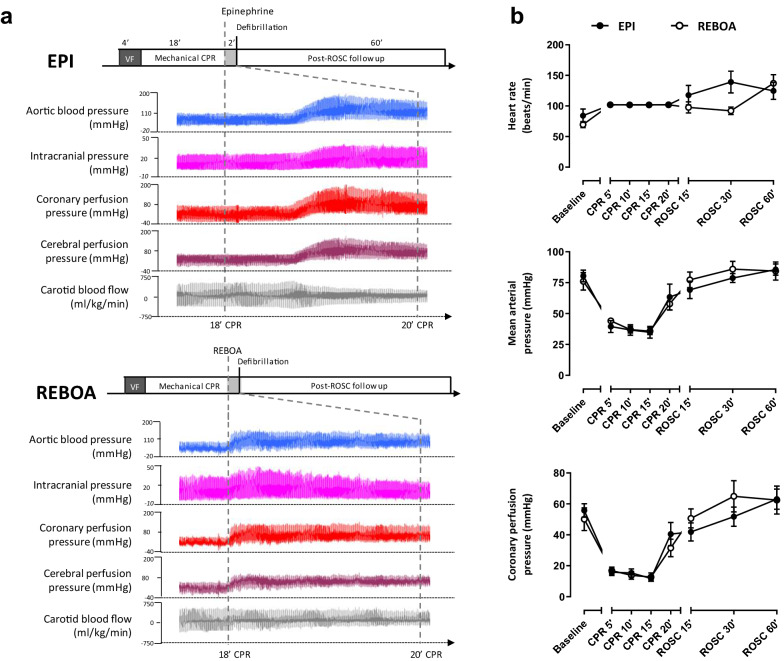
Typical waveforms of hemodynamic parameters, heart rate, mean arterial and coronary perfusion pressure. **a** Experimental protocol and typical hemodynamic tracings in two animals before and after either epinephrine (EPI, upper panel) administration or resuscitative endovascular balloon occlusion of the aorta (REBOA, lower panel), respectively. **b** Heart rate, mean arterial pressure and coronary perfusion pressure during the protocol (i.e., at baseline, during CPR and after ROSC). *N* = 6 in both groups; CPR, cardiopulmonary resuscitation; ROSC, return of spontaneous circulation

After surgical preparation and stabilization, ventilation was interrupted, and ventricular fibrillation (VF) was induced by using a pacemaker catheter introduced into the right ventricle through the venous femoral sheath. VF was left untreated for 4 min, after which conventional CPR was initiated using an automated device (LUCAS III, Stryker Medical®, Kalamazoo, MI, USA), at the rate of 100 compressions/min. Mechanical ventilation was simultaneously resumed (10 breaths/min; tidal volume = 6 ml/kg; FiO_2_ 100%; PEEP = 0 cmH_2_O). As illustrated in Fig. 1, animals were randomized to one of the 2 treatment groups, i.e., REBOA or Epinephrine (EPI). In REBOA, the REBOA Catheter (ER-REBOA, Prytime Medical®, Boerne, TX, USA) was inserted into the arterial femoral sheath and left deflated until necessary. The balloon was placed in zone I (i.e., in the thoracic descending aorta) by using anatomical landmarks. Correct placement of the REBOA was checked by post-mortem examination. After 18 min of CPR, the balloon was inflated and remained so until ROSC was obtained. In EPI, animals were given a 0.5 mg epinephrine intravenous bolus after 18 min of CPR, and then every 4 min if necessary, until ROSC. Defibrillation attempts started after 20 min of CPR, i.e., 2 min after epinephrine administration or balloon occlusion. After ROSC, mechanical chest compressions were interrupted, and initial mechanical ventilation parameters were resumed. In REBOA, the balloon was immediately deflated after ROSC. Norepinephrine was administered with the objective to maintain MAP above 60 mmHg. In the absence of ROSC, CPR was interrupted after a total duration of 30 min.

Hemodynamic and respiratory parameters were monitored during the entire protocol. Arterial blood gases were performed at baseline, after 15 min of CPR, and after 15 and 60 min of ROSC.

### Statistical analysis

Data were expressed as mean ± SEM. Body weight and number of shocks were compared between the groups using a Student’s t-test. Hemodynamic parameters were compared between groups using a Student’s t-test at baseline and a two-way ANOVA for repeated measures during CPR (at 5, 10, 15 and 20 min of CPR) and after ROSC (at 15-, 30- and 60-min post-ROSC), with a Holm–Sidak post hoc analysis. Post hoc comparisons were only made between groups at each time-point (REBOA vs EPI), but not between time-points. Significant differences were determined at *p* ≤ 0.05.

## Results

**Fig. 2:**
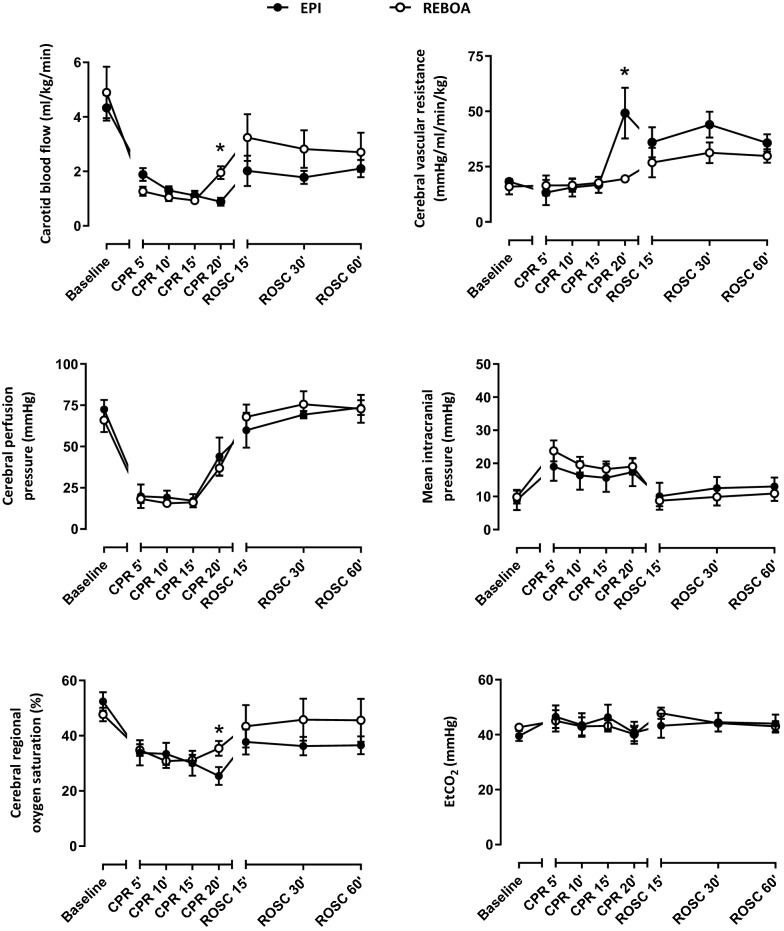
Carotid blood flow, cerebral vascular resistance, cerebral perfusion pressure, mean intracranial pressure, cerebral regional oxygen saturation and end-tidal CO2 throughout experimental protocol. * *P* < 0.05 between EPI and REBOA group; CPR, cardiopulmonary resuscitation; ROSC, return of spontaneous circulation. *N* = 6 in both groups, except for cerebral oximetry, which was only available in 5 animals in the Epi vs 6 in the REBOA group, respectively. Statistical comparisons were only made at each time-point between groups, but not between time-points

**Fig. 3:**
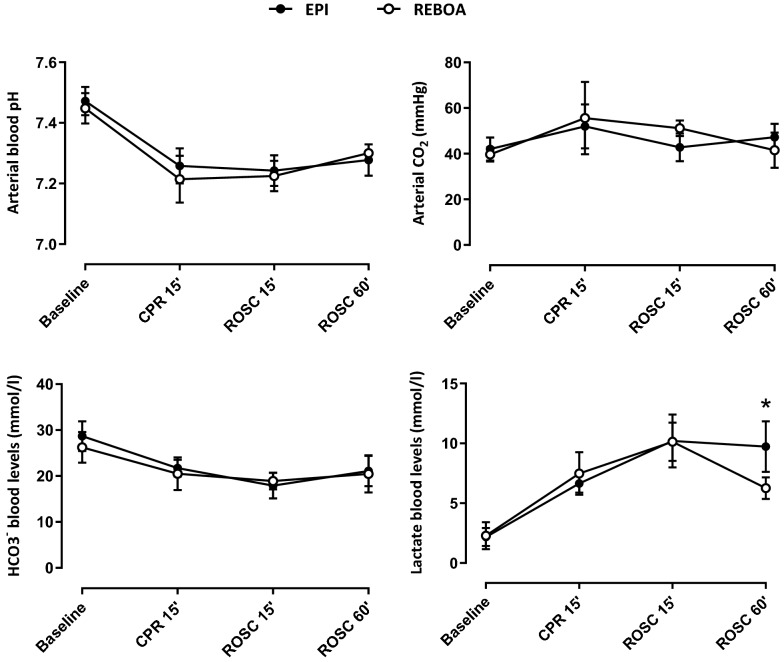
Blood gases and lactates blood levels throughout experimental protocol. *N* = 6 in both groups; * *P* < 0.05 between EPI and REBOA group; CPR, cardiopulmonary resuscitation; ROSC, return of spontaneous circulation. Statistical comparisons were only made at each time-point between groups, but not between time-points

The experimental protocol is illustrated by Fig. 1a. Twelve swine were included in the study, i.e., 6 in both EPI and REBOA groups, respectively. Body weights were similar in both groups (27.7 ± 0.7 and 27.0 ± 0.9 kg, respectively). As illustrated in Figs. 1b, 2 and 3, hemodynamic and biochemical parameters were similar between groups at baseline.

### Hemodynamics during CPR

As illustrated in Figs. 1b, 2 and 3, MAP, CoPP, ICP, CePP and CVR were similar in both groups during CPR before balloon occlusion or epinephrine administration. Figure 1a illustrates the typical effect of epinephrine administration and REBOA inflation on hemodynamic parameters. As shown by the tracing, the effects of REBOA were immediate, whereas they were delayed by at least 60 s after epinephrine administration.

Both epinephrine administration and balloon occlusion after 18 min of CPR led to an increase in MAP, CoPP and CePP between CPR 15 and 20 min. As an example, CoPP rose from 12 ± 2 to 41 ± 8 mmHg in EPI, and 13 ± 3 to 32 ± 6 mmHg in REBOA, respectively. However, CBF increased after REBOA, whereas it decreased after epinephrine administration. CVR also drastically increased in the EPI group, whereas it remained stable in the REBOA group. The differences in CBF and CVR between the 2 groups were statistically significant at CPR 20’ (+ 125 and -52% with REBOA vs EPI, respectively). Importantly, cerebral regional oxygen saturation was also significantly improved with REBOA as compared to EPI at CPR 20 min.

### Hemodynamics after ROSC

Five animals were successfully defibrillated in each group with a mean number of 1.3 ± 0.2 and 2.0 ± 0.4 shocks (*p* = 0,217) in the Epi and REBOA groups, respectively. The 2 animals that did not achieve ROSC (one in each group) had a CoPP which remained below 20 mmHg during CPR despite repeated doses of epinephrine or REBOA inflation (i.e., 16 and 11 mmHg at CPR 20’, respectively).

During post-ROSC follow-up, MAP and CoPP did not differ between the groups (Fig. 1b). Norepinephrine rates of administration were not significantly different between groups (e.g., 1.8 ± 0.7 and 1.1 ± 0.3 µg/kg/min at 60 min after ROSC in EPI and REBOA, respectively). As illustrated in Fig. 2, cerebral hemodynamic parameters (CBF, CVR, CePP, ICP) after ROSC were also similar between the groups. CBF had a tendency to be higher in the REBOA group, but the difference was not significant between groups. A similar trend was observed for cerebral regional oxygen saturation evolution in the REBOA group as compared to EPI.

In the animals that achieved ROSC, blood pH, O_2_ and CO_2_ partial pressures and HCO_3_^−^ levels were similar between groups after ROSC. However, lactate blood levels were lower in REBOA at 60 min after ROSC (9.9 ± 1.5 vs 6.3 ± 0.5 mmol/l, in EPI and REBOA, respectively; *p* = 0.02).

## Discussion

In our experimental model of non-traumatic CA, the use of REBOA during CPR provided similar benefits than epinephrine administration on systemic hemodynamic but appeared to preserve CBF, unlike epinephrine. If obtaining good systemic hemodynamics with high CoPP is crucial to achieve ROSC, it should not be at the expense of cerebral blood flow reduction. The right balance between the need for vascular resistance increase with vasopressors, essential to achieve ROSC, and subsequent side effects on cerebral perfusion is typically hard to find during conventional CPR management. The mitigated effect of epinephrine was indeed well shown in the clinical arena, with an improvement of short-term survival but not favorable neurological outcome as compared to placebo [1].

Here, the use of zone 1 REBOA was as efficient as epinephrine administration in increasing MAP and CoPP in order to facilitate ROSC. CoPP is indeed known to be the main predictive factor of ROSC, as demonstrated by Reynolds et al. during experimental CPR [4]. The capacity of REBOA to improve CoPP and thus increase chances of ROSC has been shown before [15]. However, until now, REBOA had not been evaluated as a “substitute” for epinephrine, even in animal studies. In most cases, REBOA was used in addition to epinephrine. The objective of our study was to evaluate the sole effect of REBOA vs epinephrine administration.

Beyond its effect on CoPP and ROSC, the inflation of the REBOA in the descending aorta led to an increase in CBF, as previously observed during CPR in a cardiac arrest model in piglets [11]. On the contrary, the administration of epinephrine led to a reduction of CBF, as previously shown by Burnett et al. [5]. However, the use of CBF as a marker of cerebral blood flow should be discussed. Beyond vascularization of the brain, CBF could also reflect perfusion of the snout and skin of the pig’s face. Therefore, finding the perfect surrogate for a continuous evaluation of cerebral blood flow during CPR is difficult. Some authors suggest using cerebral perfusion pressure [16]. In our study, CePP similarly increased in both study groups, i.e., after REBOA occlusion and epinephrine administration. However, other studies support that CePP does not accurately reflect cerebral microcirculation. Indeed, Shaffner et al. [17] showed that CePP led to different cerebral blood flow and metabolism after different periods of no-flow. Cerebral oxygen saturation is then typically considered as a good alternative for the non-invasive evaluation of brain microcirculation. Importantly, this parameter was also improved during REBOA inflation as compared to EPI, in line with CBF data in the present study. One would then argue that our findings mostly evidence the deleterious effect of epinephrine on cerebral perfusion during CPR. Indeed, Ristagno et al. also showed that epinephrine decreased cerebral microvascular blood flow, reduced the number of perfused cerebral capillaries, and increased the severity of cerebral ischemia, even after ROSC in a model of non-traumatic CA [6]. These consequences were shown to be more specifically due to the α_1_-adrenergic effects of epinephrine. Wagerle et al. also showed that the vasoconstriction and increase in cerebral resistance of epinephrine was mediated by the α_1_-adrenergic receptors in newborn piglet cerebral vessels [18]. In our study, the administration of epinephrine resulted in a drastic increase of CVR, in line with all these previous findings.

Beyond the superiority of REBOA to epinephrine on cerebral hemodynamics parameters during CPR, we observed, as others before, that the effect of epinephrine administration was not immediate, but delayed by more than 30 s although it was administered directly in the femoral vein (vs peripheral vein injection in the setting of out of hospital cardiac arrest). This has been shown by Putzer et al. [19] who found that MAP and CePP increase was not only delayed, but only lasted 30 to 60 s with a decreasing magnitude of the effect epinephrine over time from one bolus to another. Conversely, the beneficial effects of REBOA were immediate upon occlusion. This has its importance in the clinical setting as occlusion of the aorta has ischemic consequences in the territories below the balloon. As we were able to show, the balloon was only occluded during a few minutes and deflated immediately after ROSC, which decreases the risks of abdominal, medullar and limb ischemia. Retrospectively, this aortic occlusion could have been even shorter. With the immediate increase in MAP and CoPP, defibrillation attempts could have been made as soon as the REBOA was inflated (as compared to 2 min later as imposed by the study protocol). Despite this occlusion, lactate levels were significantly lower in REBOA at the end of our experiment. Conversely, an increase in lactate levels after epinephrine administration during CPR was previously observed by Ristagno et al. [6], suggesting that epinephrine altered microcirculation and increased tissue ischemia even after ROSC. This could explain the persistent effects of EPI on increased CVR as well as decreased CBF and cerebral oximetry observed in our study. Indeed, although the difference was not significant, CBF remained higher in REBOA even after the balloon was deflated.

### Clinical perspectives

As recently summarized by Nowadly et al., the use of REBOA in the clinical setting as a treatment for non-traumatic CA is definitely discussed [9], although many challenges remain especially for OHCA. It is important to emphasize that occlusion level of the balloon is important. Zone 1 REBOA (in the thoracic descending aorta) increases arterial pressures (especially diastolic pressure) which is not the case of Zone 3 (infrarenal aorta) placement of the REBOA [20, 21]. Even in zone 1, placing the REBOA below the heart (Zone 1-C) instead of at heart level (Zone 1-B, as confirmed by fluoroscopy) has been shown to be more efficient [22]. With this in mind, it could of course be argued that the use of REBOA is more complicated than epinephrine administration. However, it has also been shown recently that implementation of the REBOA during CPR is feasible. Indeed, the REBOA catheter was successfully placed during CPR in 9/15 patients with a median time of insertion of 9 min 30 s in the study by Levis et al. [13]. Moreover, Brede et al. have shown the possibility of implementing REBOA during CPR in the prehospital setting [23]. Depending on the system of care, REBOA implementation could be considered as a second-line therapy after failure to obtain ROSC with the first few boluses of epinephrine. However, REBOA implementation might also be considered as a surrogate for epinephrine and used as soon as ALS team is on the scene. Further clinical research is needed. In any case, once implemented and in the absence of ROSC, REBOA can also be a bridge to ECPR for the treatment of refractory cardiac arrest.

### Limitations

Our study has several limitations. First, as mentioned previously, we recognize that carotid blood flow can be an imperfect approximation of cerebral blood flow. However, we also used cerebral oxygen saturation as a surrogate marker of cerebral perfusion. Secondly, we were not able to study long-term neurological outcome, as invasive monitoring of the animals made awakening challenging. It would have been of importance and deserves further investigation. Finally, our study did not evaluate the implementation of the REBOA in itself during CPR as the femoral sheath was already in place.

## Conclusion

In a swine model of non-traumatic CA, the use of REBOA during CPR is as efficient as epinephrine to facilitate ROSC. Unlike epinephrine, REBOA transitorily increases CBF as well as cerebral regional oxygen saturation and could avoid its cerebral detrimental effects during CPR. These experimental findings suggest that the use of REBOA could be beneficial in the treatment of non-traumatic cardiac arrest. Further research is necessary to determine the appropriate indication of REBOA in this setting.

## Data Availability

Data will be available upon request to corresponding author.
